# Inhibition of Mycotoxigenic Fungi in Different Vegetable Matrices by Extracts of *Trichoderma* Species

**DOI:** 10.3390/jof7060445

**Published:** 2021-06-03

**Authors:** Claudia Stracquadanio, Carlos Luz, Federico La Spada, Giuseppe Meca, Santa Olga Cacciola

**Affiliations:** 1 Department of Agricultural Science, Mediterranean University of Reggio Calabria, Localitá Feo di Vito, 89122 Reggio Calabria, Italy; claudia.stracquadanio@unirc.it; 2 Department of Agriculture, Food and Environment, University of Catania, Via S. Sofia 100, 95123 Catania, Italy; federicolaspada@yahoo.it; 3 Department of Preventive Medicine, University of Valencia, Av. Vicent Andrés Estellés s/n, 46100 Valencia, Spain; carlos.luz@uv.es (C.L.); giuseppe.meca@uv.es (G.M.)

**Keywords:** *Trichoderma* *asperellum*, *Trichoderma* *atroviride*, bioactive metabolites, biological control, mycotoxins

## Abstract

Post-harvest fungal diseases of plant products are a serious concern leading to economic losses and health risks. Moreover, the use of synthetic chemical fungicides to prevent these diseases is limited due to toxic residues. This study aimed at determining the effective dose of extracts of *Trichoderma* *asperellum* IMI393899 (TE1) and *Trichoderma* *atroviride* TS (TE2) in inhibiting the contamination by mycotoxigenic fungi on different plant matrices. Extracts were tested on tomatoes contaminated by *Fusarium* *verticillioides* and *Fusarium* *graminearum*, wheat contaminated by *Penicillium* *verrucosum* and maize contaminated by *Aspergillus* *flavus*. The efficacy of extracts was evaluated at two time intervals after treatment, 4 and 11 days for tomato, and 10 and 20 days for both wheat and maize. Both extracts showed a significant inhibitory activity on mycotoxigenic pathogens and significantly reduced Log CFU/g compared to the control. Moreover, the extracts reduced mycotoxin production in a dose dependent manner and with a long-lasting effect. The ochratoxin A was reduced by both extracts but only the extract TE2 was effective in reducing aflatoxins, whereas TE1 treatment increased their synthesis.

## 1. Introduction

Diseases caused by fungi are the main causes of yield losses in agriculture. Fungi are responsible for numerous plant diseases and their infections can occur in the field and affect the fruit. Often the disease in the fruit does not appear immediately in the field but during the storage and distribution processes. From contaminated fruits, the disease can spread to other fruits leading to huge losses [1]. In addition, some fungi are able to produce toxic compounds called mycotoxins. Mycotoxins are secondary metabolites produced by some species of fungi, which are consequently referred to as mycotoxigenic fungi, such as *Fusarium*, *Penicillium*, *Aspergillus* and *Alternaria* [2,3,4]. Mycotoxins are not involved in fungal development or growth. However, they do play a role in plant diseases as virulence and pathogenicity factors [5]. The proliferation of fungi and the production of mycotoxins on food and feed is always favoured by some environmental factors, such as humidity and temperature, as well as by the vegetable matrix.

Although more than 400 mycotoxins have been identified, the most studied are aflatoxins (AFs), ochratoxin A (OTA), Fusarium toxins, fumonisins (FBs), zearalenone (ZEA), trichothecenes (TCT) and deoxynivalenol (DON), which cause great health risks and economic losses [6].

Mycotoxins are very commonly found in cereals and cereal products [7]. They can also be found in dairy products, spices, nuts, coffee, vegetable oils, wine and fruit juices [8,9]. These compounds are dangerous to animal and human health as they can be lethal, carcinogenic, mutagenic, teratogenic, immunosuppressive, or interfere with hormonal processes [10,11]. Their activity depends on the type of toxin and their concentration in food. Mycotoxins can be produced before and after harvest, and their levels may increase during post-harvest, handling and storage. They can reach consumers either through direct contamination of plant materials or derived products or through the accumulation of mycotoxins and their metabolites in animal tissues, milk and eggs after intake of contaminated feed [12]. In addition, this hazard remains in processed foods because these metabolites are not removed by normal industrial processing, and the risk may increase if mouldy fruits or other parts of the plant are used in by-product processing [13].

Inhibition of fungal growth in crops, fresh fruit and vegetables is therefore necessary to reduce the risk of mycotoxin contamination. Fungal growth inhibition is often controlled by the use of chemical fungicides in both pre-harvest and post-harvest. The first step in controlling fungal contamination is the application of fungicides in the field. Fungicides can be also applied in post-harvest, provided that they do not adversely affect the appearance or quality of the treated produce [14].

The indiscriminate and excessive use of fungicides in crops has been indicated as one of the main causes of the development of resistant populations of pathogens, resulting in the use of higher concentrations of these antifungals and the consequent increase in toxic residues in food products [15]. Moreover, some of these compounds are non-biodegradable, so they can accumulate in the soil, water and plants, causing environmental contamination and, through the food chain, they can be hazardous to human health. Because of these undesirable effects, the need has arisen for the development of new, safe, biodegradable alternatives that are effective and economically feasible. A viable alternative appears to be the use of microorganisms and their metabolites. In particular, the genus *Trichoderma* is known and widely used as a biological control in agriculture. The efficacy of *Trichoderma* species has been demonstrated for the management of post-harvest diseases in different crops such as papaya, tomato, apple, pear, banana, mango, berries and potato [16,17,18,19,20,21,22,23,24]. Some species of this genus are able to produce antimicrobial metabolites that inhibit the growth of fungal pathogens [25,26,27].

The objective of this study was to evaluate the capacity of metabolites extracted from two *Trichoderma* species, *T*. *asperellum* (IMI 393899) and *T. atroviride* (TS), to inhibit the growth of mycotoxigenic fungi, *Fusarium verticillioides*, *F*. *graminearum*, *Penicillium verrucosum* and *Aspergillus flavus*, and the production of their mycotoxins in different plant matrices, including tomato, wheat and maize.

## 2. Materials and Methods

### 2.1. Chemical Materials

HPLC-grade methanol and acetonitrile, analytical reagent grade methanol, ethyl acetate, formic acid (99%), and dimethyl sulfoxide (99.9% DMSO) were obtained from Thermo Fisher Scientific (Loughborough, UK). Magnesium sulfate (MgSO_4_) was obtained from Thermo Fisher Scientific (Kandel, Germany). Potato dextrose agar (PDA) and Potato dextrose broth (PDB) were obtained from Thermo Fisher Scientific (Basingstoke, UK). Ultrapure water (<18 MW/cm) was obtained from a Milli-Q purification system (Millipore Corp., Bedford, MA, USA).

Aflatoxins (AFB1 and AFB2), Ochratoxin A (OTA), Fumonisins (FB1 and FB2), (Zearalenone (ZEA) and Deoxynivalenol (DON) standards were obtained from Sigma-Aldrich (St. Louis, MO, USA).

### 2.2. Fungal Strains and Culture Conditions

Two *Fusarium* strains (*F. verticillioides* ITEM 12052 and *F. graminearum* ITEM 126) and *Aspergillus flavus* ITEM 8111 were obtained from the Agro-Food Microbial Culture Collection (Bari, Italy). *Penicillium verrucosum* VTT D-01847 was obtained from the VTT Culture Collection (Espoo, Finland). Two species of *Trichoderma* (*T. asperellum* IMI 393899 and *T. atroviride* TS), characterized in previous studies [27,28], were obtained from the collection of the Molecular Plant Pathology laboratory of the Di3A, University of Catania (Catania, Italy).

The mycotoxigenic fungi were cryopreserved in sterile 30% glycerol at −80 °C, but before antifungal studies, they were defrosted and cultured in PDB at 25 °C for 48 h and inoculated on PDA plates to obtain spores. All fungal strains were maintained on potato dextrose agar (PDA) at room temperature and subcultures were made every 20 days.

### 2.3. Liquid Culture, Production and Extraction of Metabolites

Two plugs of each *Trichoderma* strain, obtained from actively growing margins of PDA cultures, were used to inoculate 750 mL flasks containing 500 mL of sterile potato dextrose broth (PDB). The liquid cultures were incubated for 30 days at 30 °C under stirring (100 rpm) [27]. The cultures were filtered under vacuum through filter paper and the filtrates stored at 2 °C for 24 h before the biphasic extraction. The culture filtrates of *T*. *asperellum* IMI 393899 and *T*. *atroviride* TS were extracted with ethyl acetate (EtOAc) three times with a final 1:1 ratio.

The combined organic fraction was dried (MgSO_4_), filtered and evaporated under reduced pressure at 35 °C. The two red-brown residues recovered were dissolved with 10% DMSO and stored at −20 °C until the subsequent analysis.

### 2.4. Inoculum Preparation

The inoculum of the pathogens consisted of a spore suspension and was prepared by suspending the spores in buffered peptone water with 1% Tween 20. Spores were counted using the Neubauer chamber and the suspension was adjusted to the final concentration used in the assay [29].

### 2.5. Study of the Extracted Metabolites in Different Matrices

In the search for alternative antifungals to be applied in vegetable matrices, one issue to consider is their efficacy in vivo. Although in vitro screening has revealed good results, in vivo vegetable matrices can interact with bioactive compounds, decreasing their efficacy.

The evaluation of the antimicrobial activity of *Trichoderma* extracts was carried out in three different vegetable matrices inoculated with different mycotoxigenic pathogens. The matrices used were tomato fruits inoculated with *F*. *verticillioides* and *F*. *graminearum*, wheat inoculated with *P*. *verrucosum* and maize inoculated with *A*. *flavus*. 

Based on the previous study in which the extracts of *T. asperellum* IMI393899 and *T. atroviride* TS were effective in vitro on the pathogens examined; the results were expressed as minimum fungicidal concentration (MFC) [27]. Three different concentrations were tested: the MFC, and the double and the quadruple of MFC (MFCx2 and MFCx4). The concentrations are reported in Table 1.

#### 2.5.1. Tomato Fruits

The evaluation of the antifungal activity of *T*. *asperellum* (TE1) and *T*. *atroviride* (TE2) extracts against the two *Fusarium* species was carried out on tomato fruits. The cherry tomatoes were obtained from the supermarket chain Mercadona (Valencia, Spain). The fruits were initially washed under running water, sterilised for 2 min in 2% sodium hypochlorite and then rinsed with sterile water. Wet fruits were placed in plastic trays (sterilised with 70% ethanol and under UV) and dried for 2 h under a laminar flow cabinet (Telstar MH 100, Terrassa, Spain). The fruits were wounded with a sterile needle and 20 µL of a suspension of spores of the pathogen at a concentration of 10^4^ spores/mL was inoculated onto the wound; subsequently, the drop was dried for 1 h. Finally, tomato fruits were treated with 20 µL of TE1 or TE2 at three different concentrations (MFC, MFCx2, MFCx4) in the same wound where the pathogen inoculum had been applied. The MFC concentrations for both *Fusarium* species were 0.78 and 1.56 mg/mL of TE1 and TE2, respectively. The treated tomato fruits were dried under a laminar flow cabinet until the droplet was completely dry and the closed trays were incubated at room temperature. For each test, 40 tomato fruits were used. The untreated positive control was inoculated with the spore suspension of the pathogen and the negative control was the wounded fruit only (untreated and not inoculated). Fruits were monitored daily and results were evaluated at two time intervals, at 4th and 11th days of incubation. The percentage of infected fruit (% IF) was calculated based on the number of fruits with visible symptoms of infection out of the total treated fruits. The diameter of the lesions was also measured.

Subsequently, 14 tomato fruits from each test were taken randomly and eight were freeze-dried for mycotoxin analysis and six were examined for the viable microbial count.

#### 2.5.2. Wheat and Maize

The wheat (Biocesta, Valencia, Spain) was obtained from an organic supermarket and the maize was purchased from the supermarket chain Mercadona (Valencia, Spain).

The wheat and maize kernels were sterilised in an autoclave at 120 °C for 20 min. Afterwards, 5 g of wheat or maize were placed in sterile small plates (55 mm) and inoculated by spraying with 500 µL of the pathogen spore suspension (10^3^ spores/g) to wet all the surface of the kernels but avoiding excess suspension percolating. Finally, the kernels were treated by spraying with 500 µL of TE1 or TE2 at three different concentrations (MFC, MFCx2 and MFCx4, determined as mg/g of kernels). The MFCs of TE1 were 0.78 mg/mL for both *P*. *verrucosum* in wheat and *A*. *flavus* in maize, while for TE2, the MFCs were 1.56 and 6.25 mg/mL for *P*. *verrucosum* and *A*. *flavus*, respectively. After both inoculation and treatment, the kernels were dried for 2 h under a laminar flow cabinet. Plates were placed in glass flasks (sterile) and incubated at room temperature in the dark.

The control was wheat or maize inoculated with the pathogen without treatment with extracts (untreated control). For each test, 12 plates were made in order to obtain 6 repetitions per test, at 10 and 20 days after treatment. Some samples were used for viable microbial counting. Samples for mycotoxin analysis were stored at −80 °C to stop fungal growth and proliferation.

### 2.6. Viable Microbial Counting

The viable spore count test was performed using six tomato fruits at 4 and 11 days after treatment and 15 g of wheat and maize at 10 or 20 days after treatment. All vegetable matrices were homogenised with sterile buffered peptone water at a ratio of 1:10 (*w*/*v*) in a Stomacher (IUL, Barcelona, Spain) for 30 s. From the homogenate, seven serial decimal dilutions were prepared in sterile falcon (15 mL) with 9 mL of peptone water (1:10 *v*/*v* ratio). Three repetitions were performed for each sample. Subsequently, 100 µL of each tube was plated in PDA plates. The plates were incubated at 25 °C, and the number of viable colonies was counted at 72 h of incubation.

Finally, from the number of colonies per plate, the viability was calculated and expressed as Log CFU/g [30].

### 2.7. Mycotoxin Analysis

Mycotoxins were extracted using the method described by Quiles et al. (2019) [31]. Maize and wheat kernels were ground using an Oester Classic grinder (Madrid, Spain) in order to reduce particle size. Tomato fruits were frozen at −80 °C and freeze-dried before grinding. The powdered samples were weighed into 2 g of tomato fruits and 3 g of maize or wheat kernels in a Falcon tube (50 mL); to each sample, 25 mL of MeOH was added and homogenised for 3 min with an Ultra Ika T18 Ultra-turrax (Staufen, Germany). Subsequently, the extract was centrifuged at 4000 rpm for 15 min at 4 °C, and the supernatant was evaporated to dryness with a Büchi Rotavapor R-200 (Postfach, Switzerland). Finally, the dry extracts were resuspended in 2 mL methanol (HPLC-grade) and filtered through 0.22 μm before analysis. The samples were extracted in triplicate.

The HPLC system used for the chromatographic determination was an Agilent 1200 (Agilent Technologies, Santa Clara, CA, USA) equipped with a vacuum degasser, autosampler, and binary pump. The column was a Gemini NX-C18 (150 mm × 2 mm, 110 Å, and 3 m particle size; Phenomenex, Torrance, CA, USA).

The binary mobile phases consisted of water (A) and acetonitrile (B) with 0.1% *v*/*v* formic acid. The initial gradient of the mobile phase was 5% B and was increased to 95% B over 30 min. It was decreased to 5% B in 5 min, and then maintained for 3 min. The flow rate was maintained at 0.3 mL/min and 10 µL of each sample was injected.

Mass spectrometry (MS) analysis was performed using a Q-TOF-MS (6540 Agilent Ultra High Definition Accurate Mass, Santa Clara, CA, USA), equipped with an Agilent Dual Jet Stream electrospray ionisation (Dual AJS ESI, Santa Clara, CA, USA) interface in negative ion mode for the FB1, FB2, DON and ZEN, and in positive ion mode for OTA, AFB1 and AFB2. The MS range *m*/*z* was 100–1100 and the MS/MS range *m*/*z* was 50–800. The parameters were as follows: drying gas flow (N_2_), 5.0 L/min; nebulizer pressure, 60 psig; gas drying temperature, 325 °C; capillary voltage, 3.5 kV; and fragmentor voltage, 175 V. Targeted MS/MS experiments were carried out using collision energy values of 10, 20, and 40 eV. Integration and data elaboration were managed using MassHunter Qualitative Analysis software B.08.00 (Agilent, Santa Clara, CA, USA). The analysis was carried out in triplicate.

### 2.8. Statistical Analysis

Statistical analysis of data was carried out using IBM SPSS Statistics version 23.0. Data were expressed as mean ± SE of different experiments. Differences between groups were statistically analysed with one-way ANOVA followed by the Turkey HDS post-hoc test for multiple comparisons. The difference level of *p* < 0.05 was considered statistically significant.

## 3. Results and Discussion

### 3.1. Tomato Fruits Bio-Preservation

**Figure 1:**
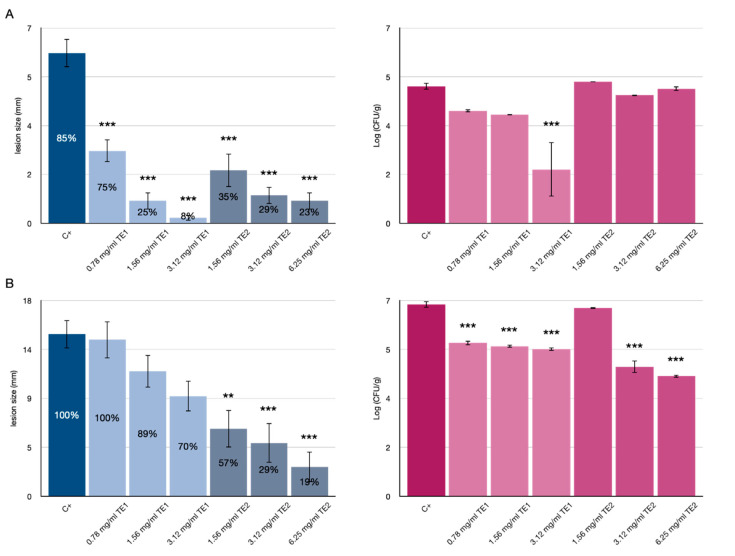
Effects of TE1 and TE2 on the growth of *Fusarium verticillioides* in tomatoes. Results expressed as % infected fruit (IF), lesion size (mm) and microbiological count (log10 CFU/g) at 4 days (**A**) and 11 days (**B**). Statistically significant differences for each treatment are indicated by ** *p* < 0.01, *** *p* < 0.001. Results are expressed as mean ± standard error.

**Figure 2:**
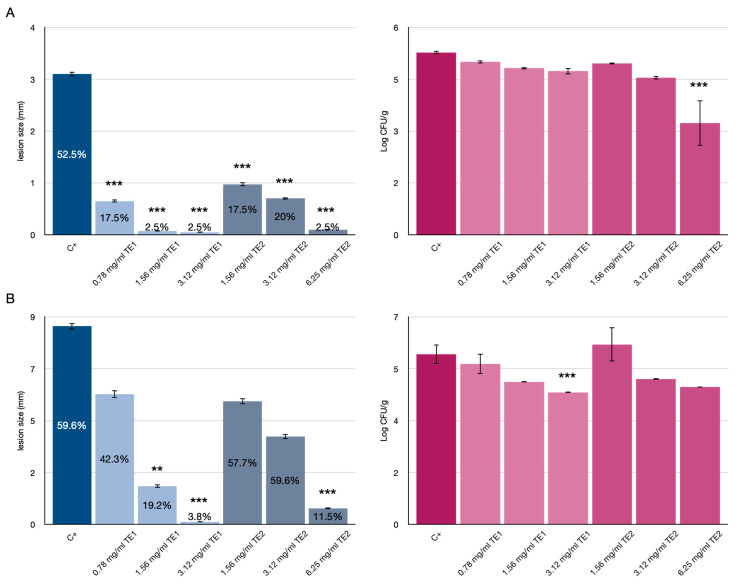
Effects of TE1 and TE2 on the growth of *Fusarium graminearum* in tomatoes. Results expressed as % infected fruit (IF), lesion size (mm) and microbiological count (log10 CFU/g) at 4 days (**A**) and 11 days (**B**). Statistically significant differences for each treatment are indicated by ** *p* < 0.01, *** *p* < 0.001. Results are expressed as mean ± standard error.

The results of application of TE1 and TE2 extracts to tomato fruits inoculated with *F. verticillioides* and *F*. *graminearum* are shown in Figure 1 and Figure 2, respectively.

At 4 days of storage, tomato fruits inoculated with *F*. *verticillioides* and treated with the extracts showed an increase in shelf-life compared with the untreated control. The untreated control had a % of IF (Infected Fruit) of 85% and 100% at 4 and 11 days of storage, respectively. Tomatoes treated with the two extracts maintained a lower % IF than the untreated control. Notably, TE2 was effective at the lowest concentration (1.56 mg/mL) with 35% IF and showing treatment persistence even at 11 days of storage with 57% IF. At 4 days, TE1 was effective at the highest concentrations with 25% IF at 1.56 mg/mL and 8% IF at 3.12 mg/mL, whereas at 11 days it was unsatisfactory with only 70% IF even at the highest concentration (3.12 mg/mL). 

These results were consistent with the analysis of lesion diameter, showing a significant reduction compared to the untreated control at all concentrations tested, with the only exception of TE1 at 11 days, which showed no significant reduction in lesion diameter compared to the control (Figure 1B). The results of the viable count analysis expressed in Log CFU/g revealed that the treatments were effective over a long time, showing a significant inhibition, compared to the control with differences at 10 days and at the highest concentration of 1.61 and 2.58 CFU/g for TE1 and TE2, respectively. Although the TE1 extract did not show clear efficacy in reducing % IF in the long term (11 days), it was effective in reducing the Log CFU/g at the highest concentration (3.12 mg/mL) at 4 days of storage and at all concentrations tested at 11 days of storage. The TE2 extract showed a clear efficacy in reducing % IF at 4 and 11 days; moreover, it maintained the Log CFU/g values stable at 4 and 11 days, being significantly (*p* < 0.001) effective in the long term compared to the control.

**Figure 3:**
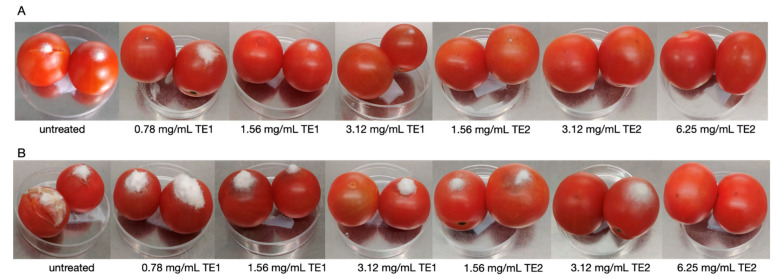
Mycelium growth of *Fusarium verticillioides* on tomato treated with TE1, TE2 and untreated at (**A**) 4 and (**B**) 11 days incubation.

**Figure 4:**
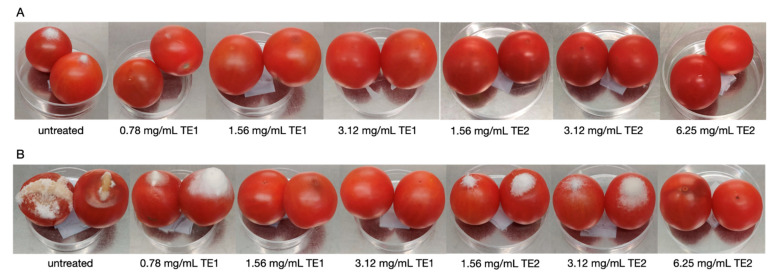
Mycelium growth of *Fusarium graminearum* on tomatoes treated with TE1, TE2 and untreated at (**A**) 4 and (**B**) 11 days incubation.

The differences in effectiveness and persistence between the two extracts could be explained by assuming they have different mechanisms of action as indicated by the different growth pattern of the mycelium on treated tomato fruits over time. Very probably the substances present in the extracts may act as elicitors, inducing the synthesis of pathogenesis-related (PR) proteins or other metabolites in tomatoes. Adss et al. (2017) [32] demonstrated that postharvest treatment with salicylic acid (SA) and H_2_O_2_ as elicitors induced the synthesis of defense-related proteins, resulting in reduced incidence of tomato rot caused by *Alternaria solani*. The TE2 extract showed a greater persistence over time, as indicated by the reduction in IF % and lesion diameter. In this case, the extract might have acted by preventing the sporulation of the pathogen on the surface of the fruit (Figure 3 and Figure 4). 

Conversely, the TE1 extract showed a lower persistence on the fruit. Nevertheless, it could have induced the biosynthesis and activation of substances or proteins with antimicrobial activity in the fruits. Yao et al. (2005) [33] observed that post-harvest treatment of sweet cherry fruit with salicylic acid (SA) or methyl jasmonate (MeJA) induced β-1,3-glucanase and peroxidase (POD) activities in early storage as a defense response, but it did not reduce the incidence of brown rot caused by *Monilinia fructicola*.

Tomatoes treated with the extracts and inoculated with *F*. *graminearum* showed a significant increase in shelf-life at 4 and 11 days, showing both lower IF % and lesion diameter with respect to the control (Figure 2). 

However, the efficacy could be attributed to the low incidence of infection of this pathogen on tomato fruits, with less than 60% IF in the untreated control. Treatment with the TE2 extract at the highest concentration tested (6.25 mg/mL) showed a reduction in microbial counts at 4 days and no significantly different values at 11 days (Figure 2), whereas the TE1 extract was effective only at the highest concentration (3.12 mg/mL) and 11 days of storage.

The mycotoxins normally produced by *Fusarium* species (FB1, FB2, ZEN and DON) were not detected by HPLC-MS-Q-TOF analysis, even in the untreated controls. Tomato fruit rot caused by *Fusarium* species is an obvious problem in postharvest production loss [34], but to the best of our knowledge no study has reported the presence of mycotoxins in tomatoes produced by *Fusarium*. In the study by Haidukowski (2004) [35], the same strain, *F. graminearum* ITEM 126, showed mycotoxin production in contaminated wheat kernels. This could possibly be due to the fact that this strain does not produce mycotoxins when infecting tomatoes but only in small grain cereals and maize [36]. 

### 3.2. Bio-Preservation of Wheat

**Figure 5:**
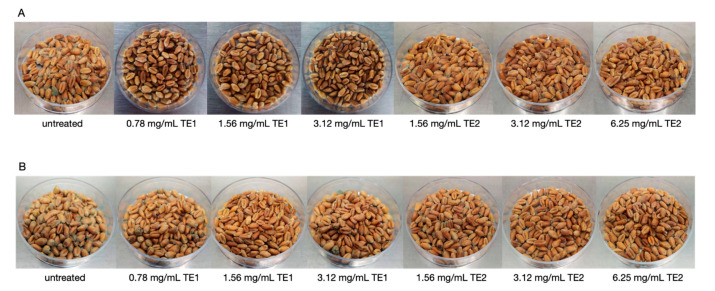
Growth of *Penicillium verrucosum* on wheat treated with TE1, TE2 and untreated, at (**A**) 10 days and (**B**) 20 days incubation.

The application of the two extracts on wheat inoculated with *P*. *verrucosum* showed significant differences compared to the untreated control. Visibly less fungal growth was observed on the wheat as the concentration increased, for both extracts at 10 and 20 days of storage (Figure 5). 

**Figure 6:**
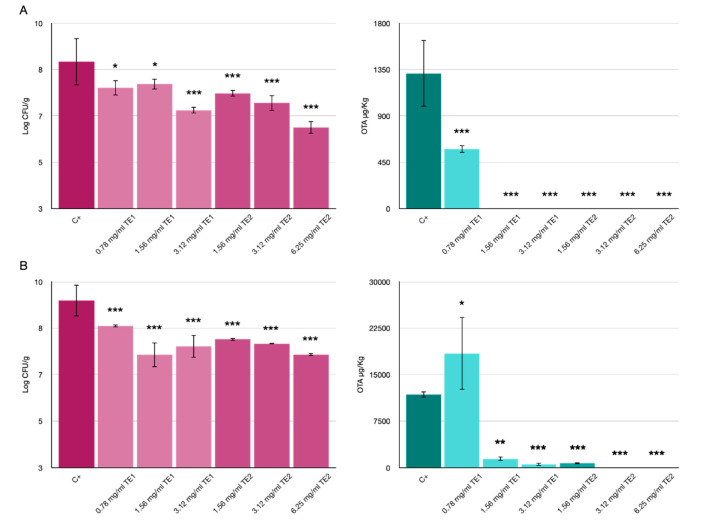
Effects of TE1 and TE2 on the growth of *Penicillium verrucosum* in wheat. Results expressed as microbiological count (log10 CFU/g) and OTA concentration detected (µg/kg) at 10 days (**A**) and 20 days (**B**). Statistically significant differences for each treatment are indicated by * *p* < 0.05, ** *p* < 0.01, *** *p* < 0.001. Results are expressed as mean ± standard error.

Mycelium growth in the untreated control was observed after 3 days of storage, while at 7 days it was observed only in the treatments at the lowest concentrations, and no growth of *P*. *verrucosum* was observed until day 10 of storage only in the case of the highest concentration of TE1 (Figure 5A). The same results were confirmed by the vital units count analysis, which showed a reduction in the number of colonies (Figure 6A). 

The results of the analysis of the OTA produced showed a reduction at both 10 and 20 days of storage (Figure 6). In particular, at 10 days, OTA was not detected in all treatments except for the treatment with TE1 at the lowest concentration (0.78 mg/mL), but in this case the OTA produced, with a value of 579 µg/kg, was lower than the untreated control (1312 µg/kg). While at 20 days, only the treatments with TE1 at the highest concentrations (3.12 and 6.25 mg/mL) showed a significant reduction with values less than 1300 µg/kg compared to the untreated control (11,770 µg/kg); while the lowest concentration of TE1 seems to have increased OTA production by *P*. *verrucosum*, probably due to the stress induced by the extract. Oddly, some fungicidal substances can stimulate the production of mycotoxins and pose a threat because of the healthy visual appearance of crops contaminated by mycotoxins. The application of fungicide azoxystrobin, e.g., in wheat contaminated by *Fusarium* spp., increased the production of the mycotoxin DON [37]. 

TE2 extract at all concentrations tested induced a reduction of OTA. In particular OTA was detected only in the treatment at the lowest concentration (1.56 mg/mL) after 20 days of storage but at a significantly lower level compared to the untreated control.

### 3.3. Bio-Preservation of Maize

**Figure 7:**
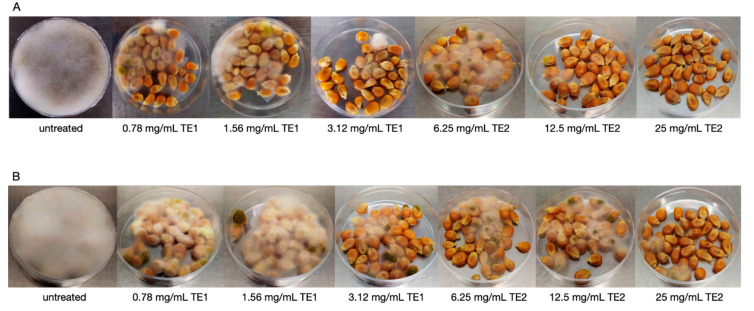
Growth of *Aspergillus flavus* on maize treated with TE1, TE2 and untreated, at (**A**) 10 days and (**B**) 20 days incubation.

Treatment with TE1 and TE2 extracts in maize inoculated with *A*. *flavus* resulted in a drastic reduction of mycelium growth, which could be clearly noticed by visual inspection. The untreated control already showed an abundant aerial mycelium on the whole plate after three days of storage. In maize treated with TE1 and TE2 at various concentrations, a stunted mycelium growth was observed at seven and ten days of storage. The abundance of aerial mycelium was inversely correlated to the concentration of the extracts (Figure 7).

**Figure 8:**
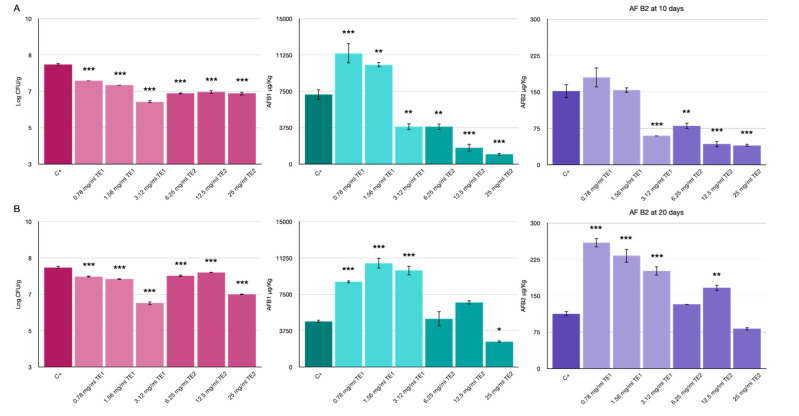
Effects of TE1 and TE2 on the growth of *Aspergillus flavus* in maize. Results expressed as microbiological count (log10 CFU/g) and AFs concentration detected (µg/kg) at 10 days (**A**) and 20 days (**B**). Statistically significant differences for each treatment are indicated by * *p* < 0.05, ** *p* < 0.01, *** *p* < 0.001. Results are expressed as mean ± standard error.

A reduction in the viability of *A. flavus* was observed, with significantly lower Log CFU/g values compared with the untreated control. In particular, treatment with TE1 at the highest concentration showed a good control that was maintained up to 20 days after treatment. At 10 days, the TE2 treatments showed similar values for all three concentrations (Figure 8A); at 20 days, only the highest concentration of TE2 showed stable efficacy, while for the other two concentrations an increase in Log CFU/g was observed (Figure 8B). 

The results of the analysis of aflatoxins produced by *A*. *flavus* (AFB1 and AFB2) are shown in Figure 8. At 10 days, treatments with TE2 at all concentrations tested showed a significant reduction in AFB1 and AFB2, with values <3000 µg/kg for AFB1 and <80 µg/kg for AFB2, compared to the untreated control (7174 and 152 µg/kg of AFB1 and AFB2, respectively); only the highest concentration of TE1 (3.12 mg/mL) showed a reduction in AFB1 (3857 µg/kg) and AFB2 (59 µg/kg), while at the other two concentrations a significant increase in AFs was observed compared to the control. At 20 days, only the treatment with TE2 at the highest concentration showed a reduction in the production of mycotoxins; for all other treatments, an increase in the production of AFs was observed, which shows that the stress induced by the treatments stimulates the fungus to produce mycotoxins as a defense mechanism. However, the use of higher concentrations may inhibit viable growth and consequently mycotoxin production.

Partial inhibition of fungal growth cannot be correlated with the inhibition of mycotoxin production because this fungistatic activity may trigger secondary metabolism as a stress response. Previous studies have shown that contact with plant extracts or essential oils can enhance aflatoxin production by fungi. dos Santos Oliveira and Furlong (2008) [38] observed that aflatoxin B1 production by *A. flavus* was inhibited in the presence of methanolic extracts of banana pulp and orange peel, aubergine and potato pulp. However, these authors found that in the presence of banana pulp and potato pulp extracts, *A. flavus* produced aflatoxin B2, which was not detected in the control.

Prakash et al. (2010) [39] reported that the production of AFB1 from an *A. flavus* strain treated with *Piper betle* var. *magahi* essential oil at low concentration (0.1 µL/mL) was higher than the control. At a higher concentration of essential oil, the inhibitory effect on aflatoxin was observed, and complete inhibition was observed at 0.6 µL/mL of essential oil. These authors suggested that low doses of fungicides induced a certain stress condition that might have been responsible for increased production of mycotoxins as a defense mechanism.

## 4. Conclusions

The search for alternative antifungal substances is of great concern to the agricultural sector, mainly because of the substantial post-harvest losses occurring due to fungal contamination. Meanwhile, environmental protection agencies and organizations are expressing concern about the widespread use of synthetic fungicides that contaminate soil and water, and can reach and be harmful for the consumer through residues on food. The possibility of using naturally extracted compounds to control fungal and mycotoxin contamination is a promising alternative [40,41]. There is a rich literature on antifungal activity of plant extracts [42,43,44,45] or the application of *Trichoderma* species as biological control agents of fungal plant diseases [26,46,47]. In agreement with our results, the antifungal activity of *Trichoderma* extracts was demonstrated by other authors who envisaged these natural substances as an alternative to synthetic fungicides [48,49]. However, the application of *Trichoderma* extracts to prevent post-harvest plant or plant product diseases is a new frontier of research in the field.

When a new antifungal is to be tested, it is important to keep in mind the complexity of the food matrices and the behaviour of microorganisms when exposed to the new compound. The results obtained in this study show that metabolites extracted from *T. asperellum* and *T. atroviride* can be used in the prophylaxis of post-harvest diseases and are also able to increase the shelf-life of the produces and inhibit the production of mycotoxins. In particular, the *T. atroviride* extract showed a long-lasting efficacy and reduced both fungal growth and mycotoxin production.

Further studies, including the evaluation of toxicological implications and the development of extraction and application procedures in order to avoid the use of potentially toxic excipients or solvents such as DMSO are needed to establish whether these extracts can be used for post-harvest treatments. Another aspect that deserves to be investigated is whether a synergistic use of both extracts is possible, which could allow a reduction in the application doses.

## Figures and Tables

**Table 1:** Concentrations of extracts TE1 and TE2. Three different concentrations were tested: the MFC as determined in a previous study [27], the double (MFCx2) and the quadruple (MFCx4) of MFC. * The concentration of extracts as mg/mL for tomatoes and mg/g for maize and wheat.

	* Extract (mg/mL or mg/g)					
	TE1			TE2		
Strain	MFC	MFCx2	MFCx4	MFC	MFCx2	MFCx4
*Fusarium verticillioides*	0.78	1.56	3.12	1.56	3.12	6.25
*Fusarium graminearum*	0.78	1.56	3.12	1.56	3.12	6.25
*Penicillium verrucosum*	0.78	1.56	3.12	1.56	3.12	6.25
*Aspergillus flavus*	0.78	1.56	3.12	6.25	12.5	25

## Data Availability

The data presented in this study are available on request from the corresponding author.

## References

[1] Dutot M., Nelson L.M., Tyson R.C. (2013). Predicting the Spread of Postharvest Disease in Stored Fruit, with Application to Apples. Postharvest Biol. Technol..

[2] Stankovic S., Levic J., Petrovic T., Logrieco A., Moretti A. (2007). Pathogenicity and Mycotoxin Production by Fusarium Proliferatum Isolated from Onion and Garlic in Serbia. Eur. J. Plant Pathol..

[3] Somma S., Petruzzella A.L., Logrieco A.F., Meca G., Cacciola O.S., Moretti A. (2014). Phylogenetic Analyses of Fusarium Graminearum Strains from Cereals in Italy, and Characterisation of Their Molecular and Chemical Chemotypes. Crop Pasture Sci..

[4] Aloi F., Riolo M., Sanzani S.M., Mincuzzi A., Ippolito A., Siciliano I., Pane A., Gullino M.L., Cacciola S.O. (2021). Characterization of Alternaria Species Associated with Heart Rot of Pomegranate Fruit. J. Fungi.

[5] Masi M., Aloi F., Nocera P., Cacciola S.O., Surico G., Evidente A. (2020). Phytotoxic Metabolites Isolated from Neufusicoccum Batangarum, the Causal Agent of the Scabby Canker of Cactus Pear (*Opuntia ficus-indica* L.). Toxins.

[6] Alshannaq A., Yu J.-H. (2017). Occurrence, Toxicity, and Analysis of Major Mycotoxins in Food. Int. J. Environ. Res. Public Health.

[7] Bentivenga G., Spina A., Ammar K., Allegra M., Cacciola S.O. (2021). Screening of Durum Wheat (*Triticum turgidum* L. subsp. *durum* (Desf.) Husn.) Italian Cultivars for Susceptibility to Fusarium Head Blight Incited by *Fusarium graminearum*. Plants.

[8] Malir F., Ostry V., Pfohl-Leszkowicz A., Toman J., Bazin I., Roubal T. (2014). Transfer of Ochratoxin A into Tea and Coffee Beverages. Toxins.

[9] Drusch S., Ragab W. (2003). Mycotoxins in Fruits, Fruit Juices, and Dried Fruits. J. Food Prot..

[10] Chu F.S. (1991). Mycotoxins: Food Contamination, Mechanism, Carcinogenic Potential and Preventive Measures. Mutat. Res. Genet. Toxicol..

[11] Fung F., Clark R.F. (2004). Health Effects of Mycotoxins: A Toxicological Overview. J. Toxicol. Clin. Toxicol..

[12] Bryden W.L. (2007). Mycotoxins in the Food Chain: Human Health Implications. Asia Pac. J. Clin. Nutr..

[13] Bullerman L.B., Bianchini A. (2007). Stability of Mycotoxins during Food Processing. Int. J. Food Microbiol..

[14] Amiri A., Dugas R., Pichot A.L., Bompeix G. (2008). In vitro and in vitro activity of eugenol oil (*Eugenia caryophylata*) against four important postharvest apple pathogens. Int. J. Food Microbiol..

[15] Merrington G., Nfa L.W., Parkinson R., Redman M., Winder L. (2002). Agricultural Pollution: Environmental Problems and Practical Solutions.

[16] Valenzuela N.L., Angel D.N., Ortiz D.T., Rosas R.A., García C.F.O., Santos M.O. (2015). Biological Control of Anthracnose by Postharvest Application of Trichoderma Spp. on Maradol Papaya Fruit. Biol. Control.

[17] Dal Bello G., Lampugnani G., Abramoff C., Fusé C., Perelló A. (2015). Postharvest Control of Botrytis Gray Mould in Tomato by Antagonists and Biorational Compounds. IOBC-WPRS Bull..

[18] Quaglia M., Ederli L., Pasqualini S., Zazzerini A. (2011). Biological Control Agents and Chemical Inducers of Resistance for Postharvest Control of Penicillium Expansum Link. on Apple Fruit. Postharvest Biol. Technol..

[19] Batta Y.A. (2004). Effect of Treatment with Trichoderma Harzianum Rifai Formulated in Invert Emulsion on Postharvest Decay of Apple Blue Mold. Int. J. Food Microbiol..

[20] Mortuza M.G., Ilag L.L. (1999). Potential for Biocontrol of Lasiodiplodia Theobromae (Pat.) Griff. & Maubl. in Banana Fruits by Trichoderma Species. Biol. Control.

[21] Sangeetha G., Usharani S., Muthukumar A. (2009). Biocontrol with Trichoderma Species for the Management of Postharvest Crown Rot of Banana. Phytopathol. Mediterr..

[22] Dania V.O. (2019). Bioefficacy of Trichoderma Species against Important Fungal Pathogens Causing Post-Harvest Rot in Sweet Potato (*Ipomoea batatas* (L.) Lam). J. Bangladesh Agric. Univ..

[23] Prabakar K., Raguchander T., Saravanakumar D., Muthulakshmi P., Parthiban V.K., Prakasam V. (2008). Management of Postharvest Disease of Mango Anthracnose Incited by Colletotrichum Gleosporioides. Arch. Phytopathol. Plant Prot..

[24] Nallathambi P., Umamaheswari C., Thakore B.B.L., More T.A. (2009). Post-Harvest Management of Ber (*Ziziphus mauritiana* Lamk) Fruit Rot (*Alternaria alternata* Fr. Keissler) Using *Trichoderma* Species, Fungicides and Their Combinations. Crop Prot..

[25] Ghisalberti E.L., Sivasithamparam K. (1991). Antifungal Antibiotics Produced by *Trichoderma* spp.. Soil Biol. Biochem..

[26] Harman G.E., Howell C.R., Viterbo A., Chet I., Lorito M. (2004). Trichoderma Species—Opportunistic, Avirulent Plant Symbionts. Nat. Rev. Microbiol..

[27] Stracquadanio C., Quiles J.M., Meca G., Cacciola S.O. (2020). Antifungal Activity of Bioactive Metabolites Produced by Trichoderma Asperellum and Trichoderma Atroviride in Liquid Medium. J. Fungi.

[28] La Spada F., Stracquadanio C., Riolo M., Pane A., Cacciola S.O. (2020). Trichoderma Counteracts the Challenge of Phytophthora Nicotianae Infections on Tomato by Modulating Plant Defense Mechanisms and the Expression of Crinkler, Necrosis-Inducing Phytophthora Protein 1, and Cellulose-Binding Elicitor Lectin Pathogenic Effectors. Front. Plant Sci..

[29] Petrikkou E., Rodriguez-Tudela J.L., Cuenca-Estrella M., Gomez A., Molleja A., Mellado E. (2001). Inoculum Standardization for Antifungal Susceptibility Testing of Filamentous Fungi Pathogenic for Humans. J. Clin. Microbiol..

[30] World Health Organization (2019). Statistical Aspects of Microbiological Criteria Related to Foods: A Risk Manager’s Guide.

[31] Quiles J.M., Nazareth T.D.M., Luz C., Luciano F.B., Mañes J., Meca G. (2019). Development of an Antifungal and Antimycotoxigenic Device Containing Allyl Isothiocyanate for Silo Fumigation. Toxins.

[32] Adss I., Hamza H., Hafez E., Heikal H. (2017). Enhancing Tomato Fruits Post-Harvest Resistance by Salicylic Acid and Hydrogen Peroxide Elicitors against Rot Caused by Alternaria Solani. J. Agric. Chem. Biotechnol..

[33] Yao H., Tian S. (2005). Effects of Pre-and Post-Harvest Application of Salicylic Acid or Methyl Jasmonate on Inducing Disease Resistance of Sweet Cherry Fruit in Storage. Postharvest Biol. Technol..

[34] Bakar A.A., Izzati M.N.A., Umi Kalsom Y. (2013). Diversity of Fusarium Species Associated with Post-Harvest Fruit Rot Disease of Tomato. Sains Malays..

[35] Haidukowski M., Pascale M., Perrone G., Pancaldi D., Campagna C., Visconti A. (2005). Effect of Fungicides on the Development of Fusarium Head Blight, Yield and Deoxynivalenol Accumulation in Wheat Inoculated under Field Conditions with Fusarium Graminearum and Fusarium Culmorum. J. Sci. Food Agric..

[36] Munkvold G.P. (2017). Fusarium Species and Their Associated Mycotoxins. Mycotoxigenic Fungi.

[37] Simpson D.R., Weston G.E., Turner J.A., Jennings P., Nicholson P. (2001). Differential Control of Head Blight Pathogens of Wheat by Fungicides and Consequences for Mycotoxin Contamination of Grain. Eur. J. Plant Pathol..

[38] dos Santos Oliveira M., Furlong E.B. (2008). Screening of Antifungal and Antimycotoxigenic Activity of Plant Phenolic Extracts. World Mycotoxin J..

[39] Prakash B., Shukla R., Singh P., Kumar A., Mishra P.K., Dubey N.K. (2010). Efficacy of Chemically Characterized Piper Betle L. Essential Oil against Fungal and Aflatoxin Contamination of Some Edible Commodities and Its Antioxidant Activity. Int. J. Food Microbiol..

[40] da Cruz Cabral L., Pinto V.F., Patriarca A. (2013). Application of Plant Derived Compounds to Control Fungal Spoilage and Mycotoxin Production in Foods. Int. J. Food Microbiol..

[41] Tripathi P., Dubey N.K. (2004). Exploitation of Natural Products as an Alternative Strategy to Control Postharvest Fungal Rotting of Fruit and Vegetables. Postharvest Biol. Technol..

[42] Esserti S., Smaili A., Rifai L.A., Koussa T., Makroum K., Belfaiza M., Faize L., Burgos L., Alburquerque N., Faize M. (2017). Protective Effect of Three Brown Seaweed Extracts against Fungal and Bacterial Diseases of Tomato. J. Appl. Phycol..

[43] Jayaraj J., Wan A., Rahman M., Punja Z.K. (2008). Seaweed Extract Reduces Foliar Fungal Diseases on Carrot. Crop Prot..

[44] Pangallo S., Nicosia M.G.L.D., Agosteo G.E., Abdelfattah A., Romeo F.V., Cacciola S.O., Rapisarda P., Schena L. (2017). Evaluation of a Pomegranate Peel Extract as an Alternative Means to Control Olive Anthracnose. Phytopathology.

[45] La Spada F., Aloi A., Coniglione M., Pane A., Cacciola S.O. (2021). Natural Biostimulants Elicit Plant Immune System in an Integrated Management Strategy of the Postharvest Green Mold of Orange Fruits Incited by Penicillium digitatum. Front. Plant Sci..

[46] Woo S.L., Ruocco M., Vinale F., Nigro M., Marra R., Lombardi N., Pascale A., Lanzuise S., Manganiello G., Lorito M. (2014). Trichoderma-Based Products and Their Widespread Use in Agriculture. Open Mycol. J..

[47] Vinale F., Sivasithamparam K., Ghisalberti E.L., Marra R., Woo S.L., Lorito M. (2008). Trichoderma–Plant–Pathogen Interactions. Soil Biol. Biochem..

[48] Vinale F., Marra R., Scala F., Ghisalberti E.L., Lorito M., Sivasithamparam K. (2006). Major Secondary Metabolites Produced by Two Commercial Trichoderma Strains Active against Different Phytopathogens. Lett. Appl. Microbiol..

[49] El-Katatny M.H., Emam A.S. (2021). Control of Postharvest Tomato Rot by Spore Suspension and Antifungal Metabolites of Trichoderma Harzianum. J. Microbiol. Biotechnol. Food Sci..

